# Efficacy of multimodal rehabilitation strategies on gastrointestinal function recovery in postoperative aortic dissection patients: a narrative review

**DOI:** 10.3389/fphys.2025.1671629

**Published:** 2025-11-12

**Authors:** Jing-Hua Xiao, Jing-Xue Wei, Zi-Ting Bi, Lang Huang, Yuan-Hong Dai, Yun-Shan Zhang

**Affiliations:** Department of Rehabilitation Medicine, The First Affiliated Hospital of Guangxi Medical University, Nanning, China

**Keywords:** aortic dissection, multimodal rehabilitation strategies, recovery of gastrointestinal function, narrative review, postoperative gastrointestinal dysfunction

## Abstract

Aortic dissection is associated with significant postoperative gastrointestinal dysfunction, a common complication that adversely affects patient prognosis. Recent advancements in multimodal rehabilitation strategies have shown promise in enhancing postoperative gastrointestinal recovery, but their impact on gastrointestinal function recovery in patients after aortic dissection surgery remains unclear. The narrative review evaluates the efficacy of multimodal rehabilitation strategies on gastrointestinal function recovery in postoperative aortic dissection patients. It provides an overview of the associated pathological mechanisms and fundamental elements of multimodal rehabilitation strategies, assesses the existing clinical evidence, investigates tailored applications for specific populations, and identifies barriers and solutions to implementation. Current evidence indicates that multimodal rehabilitation strategies positively influence the recovery of gastrointestinal function in patients following aortic dissection surgery. Current evidence remains constrained by a scarcity of high-quality, large-sample randomized controlled trials specific to this population, and mechanistic understanding continues to rely heavily on extrapolation from non-cardiac surgery studies. Future efforts should integrate biomarkers, precision medicine, intelligent monitoring systems, and standardized protocols to enable large-scale multicenter randomized controlled trials and advance foundational research.

## 1 Introduction

Aortic dissection is the most common catastrophic cardiovascular arterial disease, caused by a tear in the aortic intima, which allows blood to enter the aortic media, causing intramural hematoma and splitting the aortic wall to form a true lumen and a false lumen (Zheng et al., 2017; Yin et al., 2023). Patients usually experience persistent and severe chest, back or abdominal pain, and in severe cases, life-threatening complications may occur, including heart failure, syncope or death (Mussa et al., 2016; Zhu et al., 2020). Urgency of surgical intervention is critical, given the high mortality rate associated with this condition, particularly in cases of Stanford type A aortic dissection, which involves the ascending aorta (Tolstrup et al., 2017; Kong et al., 2013). However, postoperative complications are a significant concern, especially gastrointestinal dysfunction, which occurs in 30%–50% of patients undergoing surgery for aortic dissection (Feng et al., 2022; Halm, 1996), manifesting as ileus, abdominal distension, nausea, and vomiting (Lin et al., 2024). This complication significantly prolongs hospitalization, increases healthcare costs, and elevates risks of morbidity and mortality due to reduced visceral perfusion, surgical stress, and anesthetic effects (Diaz-Castrillon et al., 2025; Achouh et al., 2006; Frankel et al., 2021). Therefore, effective management of gastrointestinal function is critical, as it may contribute to improved survival and prognosis while alleviating nursing and social burdens during recovery from aortic dissection (Zheng et al., 2017; Diaz-Castrillon et al., 2025; Zhao et al., 2012; Pouladi et al., 2025).

Currently, conventional management approaches for gastrointestinal disturbances following aortic dissection surgery frequently depend on single-agent therapies, which exhibit restricted efficacy in facilitating a prompt and thorough restoration of gastrointestinal function (Cao et al., 2020). Conversely, multimodal rehabilitation strategies (MMR), as a comprehensive interventional approach, are gaining increasing attention for improving postoperative recovery outcomes (Carli et al., 2020). This approach methodically incorporates evidence-supported strategies, like early enteral nutrition, optimization of drug interventions, and tailored physical therapy, to collaboratively foster functional rehabilitation in patients (Carli et al., 2020; Ferreira et al., 2021). MMR is regarded as an integral component of the Enhanced Recovery After Surgery (ERAS) (Ramirez et al., 2020). While both share conceptual alignment in reducing perioperative stress and advancing standardized pathways, MMR focuses more exclusively on personalized continuous rehabilitation and functional recovery during the postoperative phase rather than covering the entire perioperative process (Ferreira et al., 2021; Trocchia Mattessich, 2020). MMR may facilitate the recovery of gastrointestinal function through multiple mechanisms, including maintaining intestinal mucosal barrier integrity, modulating neuroendocrine stress responses, and enhancing physical activity, thereby alleviating ileus, improving visceral hypoperfusion, and reducing systemic inflammation (Carli et al., 2020; Ambulkar et al., 2025). This multi-dimensional coordinated interventional strategy holds potential value for patients after aortic dissection surgery, given the complexity of the procedure, frequent hemodynamic instability, high metabolic stress, and prolonged immobilization that predispose them to gastrointestinal dysfunction and delayed recovery (Achouh et al., 2006; Frankel et al., 2021; Yagdi et al., 2006). However, evidence regarding the effect of MMR on gastrointestinal function recovery in this specific population remains insufficient. Therefore, it is necessary to summarize existing knowledge in this field to guide clinical practice and inform future research.

This narrative review aims to examine the application of MMR in gastrointestinal function recovery after aortic dissection surgery. It provides an overview of related pathological mechanisms, MMR components, clinical evidence, and individualized applications for special populations, while also seeking to identify barriers and solutions to implementation, and highlight key directions for future research to advance evidence-based practice in this evolving field.

## 2 Main body

### 2.1 Pathophysiological mechanisms of gastrointestinal dysfunction after aortic dissection surgery

#### 2.1.1 Surgical trauma and inflammatory response

Surgical trauma is a significant event that initiates a complex inflammatory response, which can have profound implications for postoperative recovery, particularly in patients undergoing major surgeries such as those for aortic dissection (Fornasiero et al., 2022; Zhao et al., 2024). The mechanical damage inflicted during surgical procedures directly affects the gastrointestinal neurovascular network, disrupting the normal functioning of the gut (Helderman, 2001). This disruption is compounded by the systemic inflammatory response syndrome (SIRS) that often follows surgical trauma (Nyström, 1998). SIRS is characterized by a cascade of inflammatory mediators that can lead to increased vascular permeability, tissue oedema, and ultimately, dysfunction of the intestinal barrier (Chi et al., 2021). The release of pro-inflammatory cytokines such as interleukin-6 (IL-6) and tumor necrosis factor-alpha plays a critical role in this process, as these cytokines can exacerbate endothelial dysfunction and promote further inflammatory responses (Fornasiero et al., 2022). Elevated IL-6 levels have been demonstrated to exhibit a strong negative correlation (r = −0.71) with gastrointestinal function scores, indicating that higher IL-6 levels may hinder postoperative recovery, including prolonged gastrointestinal dysfunction (Sun et al., 2023). This cytokine not only reflects the intensity of the inflammatory response but also serves as a predictive biomarker for adverse outcomes, such as delayed recovery times (Hanna et al., 2025). The impact of surgical trauma on the gastrointestinal tract is multifaceted; it not only affects motility but also alters the gut microbiota, which can lead to dysbiosis and a compromised mucosal barrier (Wu et al., 2025; Vilz et al., 2014). Consequently, the integrity of the gut barrier is jeopardized, allowing for the translocation of bacteria and endotoxins into the systemic circulation, which can further amplify the inflammatory response and contribute to complications such as postoperative ileus (Wang Y. et al., 2024). This mucosal compromise can be assessed through biomarkers such as fecal calprotectin, a protein released by neutrophils that indicates intestinal inflammation and mucosal integrity (Jukic et al., 2021). Elevated fecal calprotectin levels have been associated with inflammatory conditions and may provide insights into gastrointestinal recovery status, with lower levels correlating with improved function scores (Gong et al., 2024).

Moreover, the neuroinflammatory reflex activated by surgical stress can lead to increased production of pro-inflammatory cytokines, resulting in a feedback loop that perpetuates inflammation and impairs recovery (Li et al., 2016; Ni and Lai, 2020; Plaschke et al., 2016). The severity of the inflammatory response can vary based on individual patient factors, surgical techniques employed, and the extent of tissue damage (Krog et al., 2016; Kehlet, 2000). For instance, minimally invasive surgical techniques have been shown to elicit a less severe inflammatory response compared to traditional open surgeries, thereby facilitating faster recovery of gastrointestinal function (Paparella et al., 2017). The systemic inflammatory response is not only a direct consequence of surgical trauma but also a reflection of the body’s attempt to restore homeostasis following the insult (Yang et al., 2020; Tang et al., 2020). However, when this response is excessive, it can lead to complications such as organ dysfunction, prolonged hospital stays, and increased morbidity and mortality rates (Wang J. et al., 2024).

In summary, the interplay between surgical trauma and the inflammatory response plays a critical role in postoperative gastrointestinal dysfunction, necessitating targeted anti-inflammatory strategies to enhance recovery (Fornasiero et al., 2022). While anti-inflammatory medications such as lidocaine have shown potential to support intestinal function recovery (Köksal et al., 2024), a recent randomized clinical trial (RCT) reported that intravenous lidocaine did not significantly improve return of gut function at 72 h after minimally invasive colon resection (Paterson et al., 2025). Therefore, a comprehensive approach that addresses surgical stress through multimodal rehabilitation strategies remains essential for optimizing postoperative gastrointestinal recovery.

#### 2.1.2 Hemodynamic changes

The hemodynamic changes that occur during aortic occlusion can significantly impact visceral organs, leading to ischemia-reperfusion injury (Zammert and Gelman, 2016; Wu et al., 2018). When the aorta is occluded, blood flow to the visceral organs is compromised, resulting in a state of ischemia (Cruz et al., 2011). This lack of blood supply can lead to cellular hypoxia, metabolic derangements, and ultimately, tissue injury (Kundu et al., 2025). The subsequent re-establishment of blood flow, while necessary for recovery, can paradoxically exacerbate the injury through a process known as reperfusion injury (Sun et al., 2025; Silvis et al., 2020). This phenomenon is characterized by oxidative stress, inflammation, and the activation of various signaling pathways that can lead to further cellular damage (Hernández-Ruiz et al., 2025; Liu et al., 2022). Studies have shown that the duration of ischemia correlates with the severity of reperfusion injury, emphasizing the importance of timely intervention during surgical procedures involving aortic occlusion (Tajeddini et al., 2024). Furthermore, the use of advanced monitoring techniques, such as near-infrared spectroscopy and blood gas analysis, can help assess the extent of ischemia and guide therapeutic strategies to mitigate injury during the perioperative period (Hu et al., 2025). Understanding these hemodynamic changes is crucial for optimizing patient outcomes following aortic surgery.

Postoperative hemodynamic instability, characterized by hypotension or hypertension, can significantly influence mesenteric blood flow and, consequently, gastrointestinal function (Abebe et al., 2022; Lonjaret et al., 2014). Hypotension, often resulting from inadequate fluid resuscitation or excessive blood loss during surgery, can lead to reduced perfusion pressure in the mesenteric circulation (Bickell et al., 1994; Akaraborworn, 2014). This reduction in blood flow can compromise the oxygen supply to the intestines, leading to potential ischemic injury and impaired gastrointestinal motility (Stefaniak et al., 2010; Martikainen et al., 2010). Conversely, postoperative hypertension can also adversely affect mesenteric blood flow by causing increased vascular resistance and potential damage to the endothelial lining of the vessels, which may lead to further complications such as mesenteric ischemia or thrombosis (Ghimire et al., 2023). Studies have demonstrated that maintaining optimal blood pressure levels postoperatively is essential for ensuring adequate mesenteric perfusion, thereby promoting gastrointestinal recovery and reducing the incidence of complications such as postoperative ileus (Mohl et al., 2025). The use of goal-directed fluid therapy and careful monitoring of hemodynamic parameters can help achieve stable blood pressure (Peng et al., 2014), thereby enhancing mesenteric blood flow and improving overall gastrointestinal function in the postoperative setting.

#### 2.1.3 Pharmacological factors

The use of opioid analgesics in the postoperative management of patients with aortic dissection is a double-edged sword, as these medications are effective for pain relief but can significantly inhibit gastrointestinal motility (Tanaka et al., 2020; Keenan et al., 2015; Wasilewski et al., 2016). Opioids exert their effects by binding to specific receptors in the central nervous system and the gastrointestinal tract, leading to decreased peristalsis and prolonged transit time (Mosińska et al., 2016; Kurz and Sessler, 2003). This inhibition of intestinal motility can result in complications such as postoperative ileus, which may delay the recovery of gastrointestinal function and prolong hospital stays (Gao et al., 2010; Doherty, 2009). Therefore, while opioids are essential for managing post-surgical pain, their potential adverse effects on gut function necessitate careful consideration of alternative pain management strategies and the implementation of multimodal analgesia to mitigate these risks.

In addition to the impact of opioids, the use of antibiotics during the postoperative period can lead to dysbiosis in the gut microbiota, further complicating gastrointestinal recovery (Baldo, , 2023). Antibiotics, while crucial for preventing infections, can disrupt the delicate balance of gut flora, resulting in a reduction of beneficial bacteria and an overgrowth of pathogenic organisms (Maier et al., 2021). This dysbiosis can manifest as diarrhea, abdominal discomfort, and an overall delay in the restoration of normal bowel function (Maier et al., 2021). The interplay between antibiotic use and gut health underscores the importance of judicious antibiotic prescribing and the potential role of probiotics or prebiotics in promoting gut microbiota recovery (Shehata et al., 2022; Older et al., 2024). Addressing these pharmacological factors is essential for optimizing gastrointestinal recovery in patients following aortic dissection surgery.

### 2.2 Core components of MMR

#### 2.2.1 Early enteral nutrition

Early enteral nutrition (EEN) plays a pivotal role in the postoperative recovery of gastrointestinal function in patients with aortic dissection (Zheng et al., 2017; Zheng et al., 2025). The implementation of low-residue formulas combined with a progressive feeding regimen has shown promising results in enhancing gut motility and reducing complications associated with delayed gastric emptying (Pan et al., 2023). Low-residue diets minimize the fiber content, which can be beneficial in the early stages post-surgery, as they reduce bowel obstruction risks while still providing essential nutrients (Levenstein et al., 1985). Gradual advancement in feeding protocols allows for careful monitoring of the patient’s tolerance, enabling healthcare providers to adjust the nutritional intake based on individual responses (Heyland et al., 2013; Rossholt et al., 2023). This tailored approach not only supports nutritional needs but also promotes the restoration of normal gastrointestinal function (Song et al., 2016). Furthermore, the incorporation of immunonutrients, such as glutamine, has been studied for its potential to enhance gut integrity and immune response (White et al., 2005; Andrade et al., 2015). Glutamine serves as a vital fuel source for enterocytes and has been linked to improved intestinal barrier function, which is crucial for preventing postoperative complications (Peng et al., 2012; Zuhl et al., 1985). The synergistic effect of low-residue diets and immunonutrients in EEN protocols could optimize recovery pathways (Pan et al., 2023; Abreu Nascimento et al., 2024), ultimately leading to better outcomes for patients recovering from aortic dissection surgeries.

#### 2.2.2 Optimization of drug interventions

The implementation of non-opioid multimodal analgesia regimens has emerged as a pivotal strategy in enhancing postoperative recovery, particularly in patients undergoing major surgeries such as aortic dissection repair (Koh et al., 2015; Heybati et al., 2023). These regimens aim to minimize the reliance on opioids, thereby reducing the associated risks of opioid-related side effects, including gastrointestinal dysfunction, which can significantly hinder recovery (Martinez et al., 2019; Zimmerman and Laitman, 2024). Multimodal analgesia typically involves the use of a combination of analgesics that act through different mechanisms, thus providing synergistic effects while minimizing individual drug dosages (Polomano et al., 2017). For instance, the use of non-steroidal anti-inflammatory drugs (NSAIDs), acetaminophen, and regional anesthesia techniques, such as nerve blocks, has been shown to effectively control pain while promoting early mobilization and gastrointestinal recovery (Kehlet, 2025). Studies have demonstrated that patients receiving multimodal analgesia report lower pain scores and experience a shorter time to first flatus and bowel movement compared to those treated with traditional opioid-based regimens (Opincans et al., 2024). Furthermore, the integration of these non-opioid strategies not only alleviates pain but also addresses the physiological stress response triggered by surgical trauma, which is known to exacerbate postoperative complications, including gastrointestinal paralysis (Luo et al., 2020). Although aortic dissection surgery is complex, time-consuming, and presents many challenges, preliminary studies have reported the application of non-opioid analgesia regimens in this population. For example, the study by Zhang et al. (Zhang Y. et al., 2025) indicated that a scheduled non-opioid analgesic regimen combining oral NSAIDs such as ibuprofen with acetaminophen is feasible in patients following aortic dissection surgery. This approach not only effectively reduces opioid consumption and lowers the risk of related adverse effects such as respiratory depression and gastrointestinal dysfunction, but also maintains satisfactory pain control. Similarly, other clinical studies (Cheng et al., 2022; Gao et al., 2022) have demonstrated that adjuvant analgesics like dexmedetomidine and ketamine can also be utilized for pain management in post-aortic dissection patients, enhancing analgesic efficacy while further reducing the incidence of gastrointestinal dysfunction. By optimizing pain management through non-opioid approaches, clinicians can significantly enhance patient outcomes, reduce hospitalization duration, and improve overall satisfaction with the surgical experience (Rech et al., 2022; Kannan et al., 2025).

The rational use of prokinetic agents, such as erythromycin, has gained attention in the context of postoperative gastrointestinal recovery, especially following major surgical interventions like aortic dissection repair (Booth et al., 2002; Bradley, 2001). Erythromycin, a macrolide antibiotic, has been identified for its prokinetic properties, which stimulate gastric motility and enhance gastrointestinal transit (Bhadra et al., 2005; Sanger et al., 2013). This effect is particularly beneficial in patients who often experience postoperative ileus, a common complication characterized by delayed gastric emptying and bowel function (Zhang and Luo, 2023). Clinical trials have indicated that the administration of erythromycin can significantly reduce the time to first flatus and bowel movement, thus facilitating quicker recovery of gastrointestinal function post-surgery (Nanthiphatthanachai and Insin, 2020). The mechanism by which erythromycin exerts its prokinetic effects involves the agonism of motilin receptors in the gastrointestinal tract, leading to increased peristalsis and improved gastric emptying (Cooper et al., 2022). However, the use of erythromycin should be judicious, considering potential side effects such as QT interval prolongation and the risk of developing antibiotic resistance (Hawkyard and Koerner, 2007). Therefore, it is crucial for healthcare providers to evaluate the appropriateness of erythromycin in the postoperative setting, balancing its benefits in promoting gastrointestinal recovery against the risks associated with its use. Furthermore, ongoing research is essential to establish optimal dosing regimens and to identify patient populations that would benefit most from prokinetic therapy, thereby enhancing the overall efficacy of multimodal rehabilitation strategies in postoperative care.

#### 2.2.3 Physical therapy

Physical therapy plays a crucial role in the postoperative recovery of patients who have undergone aortic dissection repair, particularly in enhancing gastrointestinal function (Lin et al., 2023; Carbone et al., 2024). Early mobilization is a fundamental aspect of postoperative care that has been shown to significantly promote intestinal motility (Carbone et al., 2024; Thörn et al., 2022). Engaging patients in early physical activity helps to mitigate the effects of prolonged bed rest and stimulates the gastrointestinal tract, thereby reducing the incidence of postoperative ileus, which is a common complication following abdominal surgery (Debas et al., 2025; Boden et al., 2018). Studies indicate that patients who participate in structured early mobilization protocols experience faster recovery of bowel function, shorter hospital stays, and a reduced need for pharmacological interventions to manage postoperative complications (Mao and Yang, 2023). Furthermore, the integration of physical therapy into ERAS protocols has been associated with improved overall outcomes, highlighting the importance of a multidisciplinary approach that includes physical rehabilitation as a key component of postoperative care (Draeger et al., 2021).

In addition to early mobilization, evidence supports the use of abdominal massage and electrical stimulation as effective adjunct therapies in the recovery of gastrointestinal function post-surgery (Martensen et al., 2025; Chen et al., 2015). Abdominal massage, a non-invasive technique, has been shown to enhance peristalsis and alleviate discomfort associated with postoperative bloating and constipation (Turan and Aşt, 2016). This technique can be particularly beneficial for patients who are hesitant to engage in physical activity due to pain or discomfort (Turan and Aşt, 2016). Clinical studies have demonstrated that abdominal massage can lead to significant improvements in bowel function, with patients reporting a decrease in the time to first bowel movement and an overall enhancement in gastrointestinal comfort (Ambulkar et al., 2025). On the other hand, electrical stimulation, including techniques such as electroacupuncture, has gained traction as a complementary therapy in ERAS protocols (Mao and Yang, 2023). The application of electrical stimulation has been shown to modulate gastrointestinal motility, reduce postoperative nausea and vomiting, and improve overall recovery of gastrointestinal function (Mao and Yang, 2023). This is particularly relevant for patients who may experience delayed gastric emptying or other motility disorders following major surgery (Mao and Yang, 2023; Chen, 2025).

The integration of evidence-based physical therapy modalities, including early mobilization, abdominal massage, and electrical stimulation, contributes significantly to both immediate recovery and long-term functional outcomes in post-aortic dissection patients by reducing complications and enhancing clinical recovery (Lin et al., 2023; Carbone et al., 2024; Mao and Yang, 2023). Ongoing research remains essential to further refine these multimodal rehabilitation protocols and optimize their implementation in clinical practice.

### 2.3 Clinical evidence of MMR on gastrointestinal function recovery

The integration of MMR has demonstrated significant benefits in enhancing gastrointestinal function recovery following aortic dissection surgery. Although high-quality evidence remains limited, available studies consistently support the value of structured rehabilitation protocols in improving postoperative outcomes. To provide a clearer overview of the existing clinical evidence, a summary of relevant studies is presented in Table 1.

**TABLE 1:** Summary of clinical evidence on MMR for gastrointestinal function recovery in postoperative aortic dissection patients.

Study	Study type	Sample size	Postoperative MMR	Key outcomes
Zhao and jiang. 2017	RCT	64 (IG:36; CG:28)	Early mobilization, optimized analgesia, enteral nutrition	Shorter time to bowel sound recovery, first flatus, and defecation (p < 0.05); reduced incidence of postoperative ileus
Lü et al., 2018	Cohort study	12 (IG:12)	Physical activity, nutritional support, pain management	Shorter time to bowel sound recovery, first flatus, and defecation (p < 0.05); reduced incidence of postoperative ileus
Guo et al., 2014	Cohort study	20 (IG:20)	Nutritional support, abdominal massage	Disappearance of gastric retention; rapid recovery of prealbumin levels

MMR, multimodal rehabilitation strategies; RCT, randomised controlled trial; IG, intervention group; CG, control group.

#### 2.3.1 RCT results

Evidence from an RCT conducted in 64 patients (intervention group: 36; control group: 28) revealed that a structured multimodal rehabilitation protocol including early mobilization, optimized analgesia, and enteral nutrition significantly accelerated the recovery of bowel sounds, first anal flatus, and defecation time compared to the control group (p < 0.05) (Zhao and Jiang, 2017). These improvements reflect an accelerated return to normal gastrointestinal motility, which is a critical determinant of postoperative recovery. Additionally, this intervention markedly reduced the incidence of postoperative ileus, underscoring its potential to mitigate common complications that prolong hospitalization and increase healthcare burdens (Chen et al., 2015). Despite these promising results, RCTs specifically targeting aortic dissection patients remain limited, underscoring the need for further high-quality trials to validate and refine these protocols.

#### 2.3.2 Cohort study results

Cohort studies have further elucidated the benefits of MMR on postoperative gastrointestinal recovery in aortic dissection patients. One study involving 12 patients demonstrated that a rehabilitation program incorporating physical activity, nutritional support, and pain management contributed to a significant reduction in hospital stay by an average of 3.5 days (p < 0.01), along with a more rapid recovery of prealbumin levels (Lü et al., 2018). Another study of 20 patients showed that an intervention focusing on nutritional support and abdominal massage led to the disappearance of gastric retention and similarly promoted rapid normalization of prealbumin levels (Guo et al., 2014). Prealbumin, a sensitive marker of nutritional status, is crucial for assessing protein synthesis and overall metabolic health (Zhao and Jiang, 2017). The accelerated recovery of prealbumin levels observed in these studies suggests that multimodal rehabilitation facilitates earlier restoration of nutritional homeostasis, which is essential for wound healing and comprehensive recovery (Beck and Rosenthal, 2002). Together, these findings underscore the clinical value of structured rehabilitation protocols in optimizing recovery trajectories and improving resource utilization in patients undergoing aortic dissection surgery.

### 2.4 Individualized applications for special populations

#### 2.4.1 Elderly patients after aortic dissection surgery

In the context of postoperative care for elderly patients who have undergone surgical intervention for acute type A aortic dissection, individualized pharmacological management is critical (Li W. et al., 2025). Given the unique physiological changes associated with aging, it is essential to adjust medication dosages based on the specific nutritional needs and metabolic responses of elderly patients (Forbes et al., 2012). This tailored approach ensures that the recovery process is both safe and effective, minimizing the risk of adverse drug reactions that are more prevalent in older populations due to polypharmacy and altered pharmacokinetics (Davies and O'Mahony, 2015). For instance, studies have shown that elderly patients often exhibit altered absorption, distribution, metabolism, and excretion of drugs, necessitating careful monitoring and adjustments to therapeutic regimens (Zhong et al., 2022). Furthermore, the nutritional status of these patients can significantly impact their recovery trajectory (Zhong et al., 2022). Thus, healthcare providers must assess their dietary intake and nutritional needs regularly. This individualized approach not only enhances recovery outcomes but also supports the overall wellbeing of elderly patients during their rehabilitation phase.

In addition to pharmacological adjustments, assessing the degree of frailty in elderly patients plays a pivotal role in guiding their rehabilitation intensity and activity plans post-surgery (Vigorito et al., 2017). Frailty, characterized by diminished physiological reserve and increased vulnerability to stressors, is particularly prevalent in older adults and can significantly affect postoperative recovery (Llerena et al., 2022). By evaluating frailty levels, healthcare providers can tailor rehabilitation programs that align with the patients’ functional capabilities and overall health status (Llerena et al., 2022). For example, a comprehensive geriatric assessment can identify specific deficits in strength, endurance, and mobility, allowing for the development of a personalized rehabilitation strategy that gradually increases the intensity of physical activities based on the patient’s tolerance and recovery progress (Thomas et al., 2008). Evidence suggests that targeted rehabilitation interventions can improve functional outcomes and reduce the incidence of complications such as prolonged hospital stays or readmissions (Llerena et al., 2022). Moreover, incorporating multidisciplinary teams, including physiotherapists and nutritionists, can enhance the quality of care provided, ensuring that all aspects of the patient’s recovery are addressed holistically (Field et al., 2011). Ultimately, a tailored rehabilitation strategy that considers both pharmacological management and the assessment of frailty can lead to improved postoperative outcomes and quality of life for elderly patients recovering from acute type A aortic dissection surgery.

#### 2.4.2 Complex aortic dissection cases

In the management of complex aortic dissection cases, particularly those undergoing visceral artery reconstruction, the selection of an appropriate nutritional pathway is crucial for optimizing postoperative recovery (Zhou et al., 2023). Patients with complex aortic dissection often experience significant gastrointestinal dysfunction due to the extent of surgical intervention and the potential for ischemia (De Paulis et al., 2022). Therefore, nutritional strategies must be tailored to individual needs, considering factors such as the patient’s baseline nutritional status, the extent of the surgical procedure, and the anticipated recovery timeline. Enteral nutrition is generally preferred, as it helps maintain gut integrity and function, but in cases where this is not feasible, parenteral nutrition may be necessary (Baiu and Spain, 2019). Continuous monitoring of nutritional parameters and adjusting the nutritional support based on the patient’s progress can significantly enhance postoperative outcomes, reducing complications and promoting quicker recovery of gastrointestinal function (Zeng et al., 2024).

Determining the optimal timing for interventions in patients requiring secondary surgeries is another critical aspect of improving postoperative recovery in complex aortic dissection cases (Zhang et al., 2023). Secondary surgeries may be necessitated by complications arising from the initial procedure or by the progression of the disease (Ameli-Renani et al., 2015). The timing of these interventions can significantly impact recovery trajectories. Early intervention may be beneficial in cases where complications such as graft failure or ischemia are identified, as it can prevent further deterioration of the patient’s condition (Zhang et al., 2023). Conversely, delaying surgery until the patient is more stable may also be advantageous, allowing for better overall recovery and rehabilitation (Ameli-Renani et al., 2015). A multidisciplinary approach involving surgeons, nutritionists, and rehabilitation specialists is essential to assess each patient’s unique circumstances and to devise a comprehensive plan that optimizes the timing and type of interventions, ultimately enhancing postoperative recovery and quality of life (Vendramin et al., 2023; Nienaber et al., 2016).

#### 2.4.3 ECMO-supported patients after aortic dissection surgery

The use of extracorporeal membrane oxygenation (ECMO) in patients following aortic dissection surgery, particularly in the context of postoperative complications such as cardiogenic shock, necessitates a tailored approach to nutrition and rehabilitation (Wang et al., 2019). Given the unique physiological demands of these patients, who often present with compromised cardiac function and systemic instability, individualized nutritional strategies are crucial (Guo et al., 2025). These strategies should prioritize the provision of adequate caloric intake and essential nutrients to support metabolic needs and promote recovery (Guo et al., 2025). For instance, studies have indicated that patients on VA-ECMO often require higher caloric supplementation due to increased energy expenditure associated with critical illness and the physiological stress of ECMO support (Wu et al., 2023). Moreover, the timing of nutritional interventions plays a pivotal role; early initiation of enteral feeding, when feasible, can help prevent the deterioration of gut function and minimize the risk of complications such as intestinal ischemia, which is a concern in patients with aortic dissection (Ozawa et al., 2022). Additionally, the integration of physical rehabilitation into the care plan is essential, as it aids in restoring mobility and function, which can be severely impacted by prolonged intensive care unit stays and mechanical support (Li S. et al., 2025). Rehabilitation should be initiated as early as the patient’s condition allows, focusing on gradual mobilization and exercises tailored to the patient’s capabilities, thereby enhancing overall recovery and quality of life post-discharge (Meng et al., 2024).

Monitoring for ECMO-related complications is equally critical in ensuring timely evaluation and intervention for gut function (Byun et al., 2024). Patients supported by ECMO are at a heightened risk for gastrointestinal complications, including ischemia and dysmotility, which can significantly affect their recovery trajectory (Mayer et al., 2022; Pérez et al., 2022). Regular assessments of bowel function, including monitoring for bowel sounds and the passage of flatus or stool, should be part of the routine care for these patients (Li et al., 2014). Furthermore, the use of abdominal imaging techniques may be warranted in cases where clinical signs suggest potential ischemia or other gastrointestinal disturbances (Gore et al., 2008). The implementation of a multidisciplinary approach, involving cardiologists, surgeons, nutritionists, and rehabilitation specialists, is paramount in addressing the complex needs of these patients (Tsangaris et al., 2021). This team-based strategy not only enhances the management of ECMO-related complications but also ensures that nutritional and rehabilitative goals are met, ultimately improving postoperative outcomes and reducing hospital length of stay (Meng et al., 2024). As such, the integration of personalized nutrition and rehabilitation protocols, alongside vigilant monitoring for complications, represents a comprehensive approach to the care of ECMO-supported patients following aortic dissection surgery, fostering better recovery and long-term survival rates (Hou et al., 2021).

### 2.5 Implementation barriers and solutions

#### 2.5.1 Clinical practice differences

The standardization of treatment protocols across various medical centers for postoperative gastrointestinal recovery in patients with aortic dissection remains insufficient (Carbone et al., 2024). This lack of uniformity can lead to significant variability in patient outcomes, as different centers may implement diverse rehabilitation strategies that are not based on a cohesive framework (Jesus and Hoenig, 2015). Such discrepancies can hinder the ability to assess the efficacy of multimodal rehabilitation approaches, as the absence of standardized protocols complicates comparative analyses (Vega Morales et al., 2025). Furthermore, a study assessing the awareness and adherence of healthcare personnel to established guidelines revealed that only 38% of respondents fully comply with these recommendations (Hedrick et al., 2018). This low adherence rate indicates a critical gap in the implementation of evidence-based practices, which may adversely affect patient care. It underscores the need for enhanced training and education initiatives to improve the understanding and application of clinical guidelines among medical staff, ultimately aiming to harmonize treatment approaches and optimize recovery outcomes for patients undergoing surgery for aortic dissection.

#### 2.5.2 Cost-effectiveness analysis

In the context of MMR for patients recovering from aortic dissection surgery, it is essential to conduct a thorough cost-effectiveness analysis. While MMR may initially increase upfront costs due to the comprehensive nature of the interventions involved, such as prehabilitation programs that include nutritional support, physical therapy, and psychological counseling, the long-term benefits can lead to a significant reduction in overall healthcare expenditures (Díaz-Feijoo et al., 2022; Rickard et al., 2021). However, these costs are often offset by the decreased need for extended hospital stays, reduced rates of postoperative complications, and lower incidences of readmissions, which collectively contribute to a more favorable financial outlook for healthcare systems (Ambulkar et al., 2025). The economic burden of postoperative complications can be substantial, and by investing in MMR, healthcare providers may ultimately save costs associated with managing these complications.

Moreover, the impact of insurance payment policies on the adoption and sustainability of MMR cannot be overlooked. Insurance reimbursement models that prioritize preventive care and rehabilitation services can incentivize healthcare providers to implement MMR programs more broadly. As healthcare systems increasingly shift towards value-based care, where reimbursement is tied to patient outcomes rather than the volume of services provided, the cost-effectiveness of MMR becomes even more relevant. Insurers may recognize the long-term savings associated with improved recovery trajectories and reduced complications (Skinner et al., 2024), leading to more favorable reimbursement rates for MMR initiatives. This alignment between cost-effectiveness and insurance policies can facilitate the integration of MMR into standard postoperative care protocols for patients with aortic dissection, thereby enhancing overall patient outcomes while simultaneously managing healthcare costs effectively (Ambulkar et al., 2025).

In conclusion, the economic evaluation of MMR shows that despite substantial initial investment, these strategies can yield significant long-term benefits through improved patient outcomes and cost savings. Future research should further investigate the economic sustainability of MMR protocols to support broader clinical implementation.

## 3 Discussion

This study aims to investigate the effects of MMR on gastrointestinal function recovery in patients following aortic dissection surgery. Through a review of current literature, it analyses the pathophysiological mechanisms underlying postoperative gastrointestinal dysfunction and the application of MMR in this context. Although existing evidence suggests the potential of MMR to enhance postoperative gastrointestinal recovery, considerable heterogeneity persists among studies, and the underlying mechanisms and optimal implementation strategies require further investigation.

**FIGURE 1:**
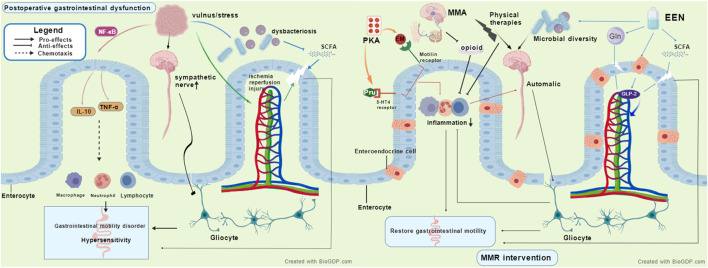
The proposed mechanisms of postoperative gastrointestinal dysfunction and how multimodal rehabilitation targets these mechanisms, MMR, Multimodal rehabilitation strategies, EEN, Early enteral nutrition, MMA, Non-opioid multimodal analgesia, PKA, Prokinetic agents, SCFA, Short-chain fatty acid.

From a pathophysiological perspective, postoperative gastrointestinal dysfunction following aortic dissection involves complex multifactorial interactions. At the cytokine level, surgical trauma activates inflammatory pathways such as NF-κB, leading to the release of pro-inflammatory cytokines including IL-6 and TNF-α (Fornasiero et al., 2022; Zhang and Zhu, 2025). These not only increase vascular permeability causing tissue edema but also disrupt intestinal epithelial tight junction proteins, impairing mechanical barrier function (Fornasiero et al., 2022; Zhang and Zhu, 2025). In terms of the enteric nervous system, surgical stress inhibits intestinal motility via sympathetic overactivation and affects enteric glial cell function, reducing the secretion of glial cell-derived neurotrophic factors, thereby compromising neuronal survival and synaptic transmission, resulting in motility disorders and visceral hypersensitivity (Xie et al., 2019). Hemodynamic changes also represent a key mechanism. Ischemia-reperfusion injury due to aortic clamping further induces cellular damage through oxidative stress and inflammatory signaling pathways, exacerbating intestinal barrier injury (Zammert and Gelman, 2016; Wu et al., 2018). Hemodynamic instability directly affects mesenteric blood flow perfusion, with hypotension reducing intestinal oxygen supply and hypertension increasing vascular resistance, collectively contributing to gastrointestinal motility disorders (Wu et al., 2018; Stombaugh and Mangunta, 2022). Additionally, disruption of the gut microbiome plays a critical role. Factors such as surgical stress and pharmacological interventions lead to reduced microbial diversity, an increase in opportunistic pathogens, and decreased synthesis of barrier-protective short-chain fatty acids, thereby compromising epithelial tight junctions, weakening the mucus barrier, and disturbing intestinal immune homeostasis (Wasilewski et al., 2016; Zhang R. et al., 2025; Das et al., 2024). Figure 1 shows the proposed mechanism of postoperative gastrointestinal dysfunction. These intertwined mechanisms collectively form the theoretical basis for MMR intervention.

MMR appears to alleviate gastrointestinal dysfunction through multiple synergistic pathways. EEN provides key nutrients such as glutamine and short-chain fatty acid precursors, maintaining intestinal epithelial energy metabolism and barrier integrity, while stimulating L-cells to secrete glucagon-like peptide-2 (GLP-2), thereby enhancing intestinal blood flow, suppressing inflammatory responses, and promoting mucosal repair (Burrin et al., 2003; Yanamaladoddi et al., 2025). EEN also helps maintain microbial diversity, increases the abundance of butyrate-producing bacteria, and strengthens the microbiota-gut-brain axis function (Cryan et al., 2019). Non-opioid multimodal analgesia reduces opioid use, mitigating their inhibitory effects on intestinal motility, and may alleviate neurogenic inflammation (Graham et al., 2025). Although certain drugs such as lidocaine show potential in anti-inflammatory and gastrointestinal function improvement (Köksal et al., 2024), their clinical efficacy remains controversial. For instance, a recent RCT did not find significant improvement in bowel function 72 h after colorectal surgery (Paterson et al., 2025), possibly due to population heterogeneity, dosing regimens, and assessment timing. Prokinetic agents like erythromycin and prucalopride enhance gastrointestinal contraction and propulsive motility via motilin receptors and 5-HT4 receptors, respectively (Spiller, 2007; Jeong et al., 2018). Physical therapies, including early mobilization, electrical stimulation, and abdominal massage, promote anti-inflammatory cytokine release, modulate autonomic nervous system balance, and enhance gastrointestinal motility through synergistic effects (Ribeiro et al., 2012; Sabino et al., 2008; Lin et al., 2025). Figure 1 depicts the potential mechanism underlying MMR intervention. However, current mechanistic evidence largely derives from animal or non-cardiac surgery studies, necessitating further validation in patients with postoperative gastrointestinal dysfunction after aortic dissection. Future research should integrate multi-omics technologies and real-time functional assessments to elucidate the molecular regulatory networks of MMR and advance the development of precision rehabilitation strategies.

This review preliminarily demonstrates that MMR can shorten the time to recovery of bowel sounds, first flatus, and hospital stay, while reducing the incidence of postoperative ileus in patients after aortic dissection surgery, consistent with findings from studies on multimodal analgesia and rehabilitation as proposed by previous studies, although these were primarily based on other surgical conditions (Ambulkar et al., 2025; Lohsiriwat, 2014; Shi et al., 2025). However, existing evidence has notable limitations, and the mechanistic foundations remain to be clarified. Few high-quality studies exist on patients after aortic dissection surgery, including only one RCT and two cohort trials, and the sample sizes are generally small. Furthermore, significant heterogeneity in intervention protocols across studies limits the comparability and reliability of results. Although early evidence indicates a role for multimodal non-opioid regimens after aortic surgery, their efficacy within a formal MMR framework remains unclear. Due to the unique complexities of aortic dissection surgery, such as its prolonged duration, significant physiological stress, and distinct pain patterns, the feasibility and effectiveness of this approach still require further study. It is important to note that the current literature on MMR in aortic dissection is largely confined to postoperative interventions. Evidence regarding structured multimodal approaches during the preoperative or intraoperative phases remains notably absent. Importantly, existing studies are mostly limited to clinical endpoint observations. The relationship between the dynamic changes in biomarkers such as IL-6 and calprotectin and rehabilitation effects remains underexplored. Moreover, there is insufficient elucidation of the mechanisms by which MMR regulates inflammatory signaling pathways, intestinal neural-immune interactions, and microbial-host interactions.

Based on the limitations of current research, future studies should prioritize large-sample, multicenter RCTs to optimize postoperative management strategies. First, molecular biomarkers should be integrated with multi-omics technologies to deeply analyze the interactions between MMR and the intestinal immune microenvironment as well as neuroendocrine networks. Besides, the implementation of precision medicine should be promoted, utilizing genetic testing for personalized medication, and optimizing gut microbial balance through microbiome intervention strategies (Nicholson et al., 2011; Trescot and Faynboym, 2014). Furthermore, intelligent monitoring systems should be developed by leveraging wearable devices to track parameters like bowel sounds in real time, and the Bayesian algorithm is combined to integrate multimodal data such as age and preoperative hypoproteinemia to achieve early prediction, realize real-time monitoring of intestinal function and early warning of complications (Shi et al., 2024). In addition, future research should focus on exploring the role of preoperative nutritional optimization and intraoperative bowel protection anesthesia protocols within the MMR framework, and integrate these elements into a broader perioperative workflow. A coordinated management strategy that integrates the preoperative, intraoperative, and postoperative phases can further optimize gastrointestinal recovery and overall surgical outcomes. Simultaneously, the synergistic effects of non-opioid analgesic strategies with other MMR components, such as early mobilization and nutritional support, should be explored, and their contribution and optimal combination in the overall rehabilitation pathway should be clarified. Finally, multidisciplinary collaboration models should be enhanced by establishing synergistic mechanisms among cardiac surgery intensive care, gastroenterology, and rehabilitation departments, adopting standardized protocols and systematizing the collection and utilization of patient-reported outcomes to form a data-driven continuous optimization system.

## 4 Conclusion

Current evidence suggests that MMR has a positive effect on gastrointestinal function recovery in patients after aortic dissection surgery, like shortening the time to recovery of bowel sounds and first flatus, reducing postoperative intestinal paralysis, and shortening hospital stay. However, the lack of high-quality, large-sample RCTs specifically in this population has resulted in insufficient evidence regarding standardized regimens and efficacy. Furthermore, the applicability of existing mechanistic evidence, largely derived from non-cardiac surgical populations, to aortic dissection patients remains unvalidated. Future efforts should integrate biomarkers, precision medicine, intelligent monitoring systems, and standardization of protocols to conduct large-scale multicenter RCTs and deepen basic research, comprehensively improving the rehabilitation quality of patients with gastrointestinal dysfunction after aortic dissection surgery.
